# Different Associations Between *CDKAL1* Variants and Type 2 Diabetes Mellitus Susceptibility: A Meta-analysis

**DOI:** 10.3389/fgene.2021.783078

**Published:** 2022-01-05

**Authors:** Qiaoli Zeng, Dehua Zou, Shanshan Gu, Fengqiong Han, Shilin Cao, Yue Wei, Runmin Guo

**Affiliations:** ^1^ Department of Internal Medicine, Shunde Women and Children’s Hospital (Maternity and Child Healthcare Hospital of Shunde Foshan), Guangdong Medical University, Foshan, China; ^2^ Key Laboratory of Research in Maternal and Child Medicine and Birth Defects, Guangdong Medical University, Foshan, China; ^3^ Matenal and Child Research Institute, Shunde Women and Children’s Hospital (Maternity and Child Healthcare Hospital of Shunde Foshan), Guangdong Medical University, Foshan, China; ^4^ State Key Laboratory for Quality Research of Chinese Medicines, Macau University of Science and Technology, Taipa, Macau SAR, China; ^5^ Institute of Neurology, Affiliated Hospital of Guangdong Medical University, Zhanjiang, China; ^6^ Department of Obstetric, Shunde Women and Children’s Hospital (Maternity and Child Healthcare Hospital of Shunde Foshan), Guangdong Medical University, Foshan, China; ^7^ Department of Medical, Shunde Women and Children’s Hospital (Maternity and Child Healthcare Hospital of Shunde Foshan), Guangdong Medical University, Foshan, China; ^8^ Department of Ultrasound, Shunde Women and Children’s Hospital (Maternity and Child Healthcare Hospital of Shunde Foshan), Guangdong Medical University, Foshan, China; ^9^ Department of Endocrinology, Affiliated Hospital of Guangdong Medical University, Zhanjiang, China

**Keywords:** type 2 diabetes mellitus, *CDKAL1*, polymorphisms, susceptibility, meta-analysis

## Abstract

**Background:** *CDK5 regulatory subunit associated protein 1 like 1* (*CDKAL1*) is a major pathogenesis-related protein for type 2 diabetes mellitus (T2DM). Recently, some studies have investigated the association of *CDKAL1* susceptibility variants, including rs4712523, rs4712524, and rs9460546 with T2DM. However, the results were inconsistent. This study aimed to evaluate the association of *CDKAL1* variants and T2DM patients.

**Methods:** A comprehensive meta-analysis was performed to assess the association between *CDKAL1* SNPs and T2DM among dominant, recessive, additive, and allele models.

**Results:** We investigated these three *CDKAL1* variants to identify T2DM risk. Our findings were as follows: rs4712523 was associated with an increased risk of T2DM for the allele model (G vs A: OR = 1.172; 95% CI: 1.103–1.244; *p* < 0.001) and dominant model (GG + AG vs AA: OR = 1.464; 95% CI: 1.073–1.996; *p* = 0.016); rs4712524 was significantly associated with an increased risk of T2DM for the allele model (G vs A: OR = 1.146; 95% CI: 1.056–1.245; *p* = 0.001), additive model (GG vs AA: OR = 1.455; 95% CI: 1.265–1.673; *p* < 0.001) recessive model (GG vs AA + AG: OR = 1.343; 95% CI: 1.187–1.518; *p* < 0.001) and dominant model (GG + AG vs AA: OR = 1.221; 95% CI: 1.155–1.292; *p* < 0.001); and rs9460546 was associated with an increased risk of T2DM for the allele model (G vs T: OR = 1.215; 95% CI: 1.167–1.264; *p* = 0.023). The same results were found in the East Asian subgroup for the allele model.

**Conclusions:** Our findings suggest that *CDKAL1* polymorphisms (rs4712523, rs4712524, and rs9460546) are significantly associated with T2DM.

## 1 Introduction

Type 2 diabetes mellitus (T2DM) is a complex disease characterized by insulin resistance in peripheral tissues and dysregulated insulin secretion by pancreatic β-cells (Li et al., 2020). The incidence of T2DM in adults has been increasing over recent decades (Yang et al., 2010; Tian et al., 2019) and is estimated to increase to over 700 million by 2045 (Saeedi et al., 2019; Li et al., 2020). T2DM is caused by genetic and environmental factors (Tian et al., 2019; Wu et al., 2014). Genetic variants are thought to be involved in the development of T2DM. Genome-wide association studies have indicated that some single nucleotide polymorphisms (SNPs) are critical risk factors for T2DM (Tian et al., 2019).

CDK5 regulatory subunit associated protein 1 like 1 *(CDKAL1)* is a crucial pathogenesis-related protein for T2DM. The *CDKAL1* gene encodes cyclin-dependent kinase 5 regulatory subunit-associated protein 1 (CDK5RAP1)-like 1. Cyclin-dependent kinase 5 (CDK5) is a serine/threonine protein kinase that contributes to the glucose-dependent regulation of insulin secretion (Li et al., 2020); therefore, it plays a critical role in the pathophysiology of β-cell dysfunction and predisposition to T2DM (Li et al., 2020; Wei et al., 2005; Ubeda et al., 2006). The associations of many SNPs in *CDKAL1* with T2DM have been examined in some meta-analyses, but no published meta-analysis has evaluated the role of *CDKAL1* rs4712523, rs4712524 and rs9460546 variants in the susceptibility to T2DM. Several studies have examined the association between *CDKAL1* polymorphisms (rs4712523, rs4712524 and rs9460546) and T2DM risk, but some findings were failed to replicate. Therefore, performing a meta-analysis is needed to evaluate the association between *CDKAL1* polymorphisms (rs4712523, rs4712524, and rs9460546) and T2DM.

## 2 Materials and Methods

This meta-analysis was conducted according to Preferred Reporting Items for Systematic Reviews and Meta-analyses (PRISMA) guidelines.

### 2.1 Literature Search

The Google Scholar, PubMed and Chinese National Knowledge Infrastructure databases were systematically searched for relevant studies using the following terms:

1 “CDKAL1” or “rs4712523” or “polymorphism” and “T2DM”;
2 “CDKAL1” or “rs4712524” or “polymorphism” and “T2DM”;
3 “CDKAL1”, or “rs9460546” or “polymorphism” and “T2DM”, respectively.

The search was performed with no date or language restrictions. All the studies were evaluated by reading the title and abstract and excluding irrelevant studies. The full texts of eligible studies were then assessed by reading the full text to confirm inclusion in the study.

### 2.2 Inclusion and Exclusion Criteria

The inclusion criteria of the studies were as follows: 1) case-control/cohort studies; 2) studies that evaluated the association between *CDKAL1* SNPs (rs4712523, rs4712524, and rs9460546) and T2DM; 3) adequate raw data or sufficient data to calculate odds ratios (ORs) with corresponding 95% confidence intervals (CIs); 4) a T2DM diagnosis based on the clinical criteria of the World Health Organization.

The exclusion criteria were as follows: 1) not a case-control/cohort study; 2) not related to *CDKAL1* SNPs (rs4712523, rs4712524, and rs9460546) and T2DM; 3) insufficient data; 4) NDM data not in Hardy-Weinberg equilibrium (HWE).

### 2.3 Data Extraction

Two authors independently extracted the following data from the included studies: first author, ethnicity, year of publication, numbers of T2DM patients and NDM controls, distribution of alleles and genotypes, and ORs with 95% CIs of the allele distribution**.**

### 2.4 Statistical Analysis

Four genetic models were evaluated in rs4712523 and rs4712524: the dominant model (GG + AG vs AA), recessive model (GG vs AA + AG), additive model (GG vs AA) and allele model (G vs A). Additionally, the allele model (G vs T) was evaluated in rs9460546. Genetic heterogeneity was estimated using Q-test and I^2^ test. Lower heterogeneity was defined as I^2^ <50% and *p* > 0.01, using the fixed effects model (Mantel–Haenszel) to calculate ORs with corresponding 95% CIs. Otherwise, the random effects model (Mantel–Haenszel) was used. The significance of the ORs was evaluated using the Z test. Begg’s and Egger’s tests were used to determine publication bias. STATA v.14.0 software (Stata Corporation, Texas, United States) was used to perform all statistical analyses.

## 3 Results

### 3.1 Study Inclusion and Characteristics

**FIGURE 1:**
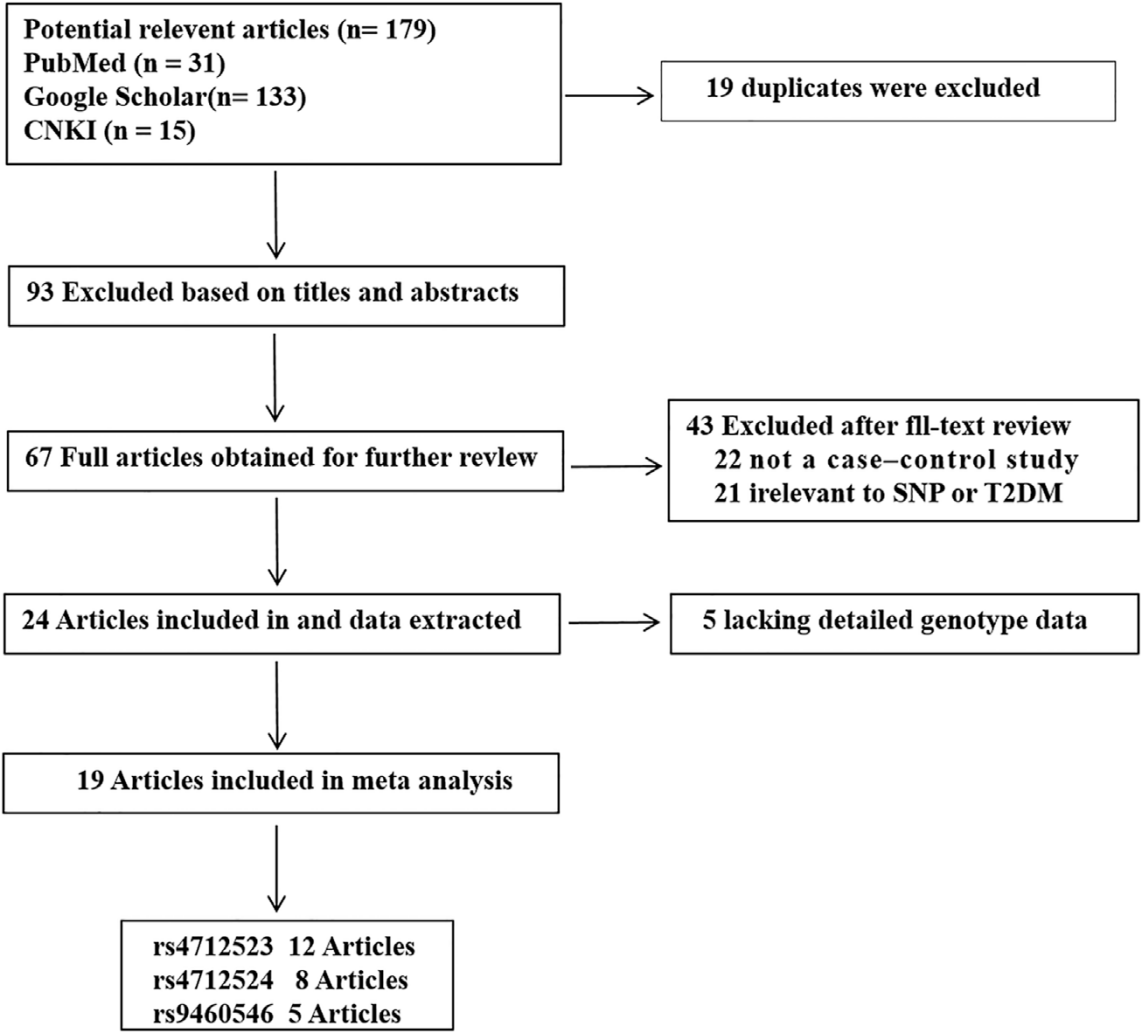
Flow diagram of the literature search and selection.

A total of 179 potential studies were searched using the inclusion and exclusion criteria. Figure 1 shows a flow chart of the study selection process. Twelve articles, including 7 in English and 5 in Chinese, had rs4712523 data. Eight articles, including 5 in English, 2 in Chinese and 1 in Russian, had rs4712524 data. Five articles, including 5 in English, had rs9460546 data. The characteristics of each included study are shown in Tables 1−3.

**TABLE 1:** Characteristics of each study included in rs4712523 of meta-analysis.

Author	Year	Ethnic	T2DM/NDM	ORs with 95% CI (G vs A)	Allele distribution				Genotype distribution					
					T2DM, n		NDM, n		T2DM, n			NDM, n		
					A	G	A	G	AA	AG	GG	AA	AG	GG
Liju et al.	2020	India	1183/1188	1.077 (0.893–1.300)	1640	726	1684	692	—	—	—	—	—	—
Tian et al.	2019	Chinese	510/503	1.420 (1.190–1.690)	508	512	588	418	131	246	133	175	238	90
Qian et al.	2019	Chinese	526/526	1.027 (0.956–1.103)	590	462	556	496	164	262	100	149	258	119
Rao et al.	2016	Chinese	458/429	0.924 (0.766–1.114)	525	391	475	383	154	217	87	138	199	92
Ren et al.	2013	Chinese	98/97	1.521 (1.018–2.273)	99	97	118	76	9	81	8	26	66	5
Li et al.	2013	Chinese	192/190	1.654 (1.237–2.212)	202	182	246	134	22	158	12	62	122	6
Lu et al.	2012	Chinese	2897/3259	1.223 (1.139–1.314)	3105	2689	3816	2702	848	1409	640	1120	1576	563
Gong et al.	2016	Chinese	91/186	1.380 (1.250–1.520)	—	—	—	—	—	—	—	—	—	—
Long et al.	2012	African Americans	1549/2722	0.960 (0.870–1.070)	—	—	—	—	—	—	—	—	—	—
Takeuchi et al.	2009	Japanese	5629/6406	1.270 (1.210–1.330)	—	—	—	—	—	—	—	—	—	—
Takeuchi et al.	2009	Europeans	14586/17968	1.120 (1.080–1.160)	—	—	—	—	—	—	—	—	—	—
Rung et al.	2009	Caucasian	180/165	1.200 (1.140–1.260)	—	—	—	—	—	—	—	—	—	—
Scott et al.	2007	Finnish	1161/1174	1.123 (1.032–1.222)	—	—	—	—	—	—	—	—	—	—

n, Number; T2DM, type 2 diabetes mellitus; NDM, Non-diabetic subject; OR, odds ratio; CI, confidence interval.

**TABLE 2:** Characteristics of each study included in rs4712524 of meta-analysis.

Author	Year	Ethnic	T2DM/NDM	Allele distribution				Genotype distribution					
				T2DM, n		NDM, n		T2DM, n			NDM, n		
				A	G	A	G	AA	AG	GG	AA	AG	GG
Liju et al.	2020	India	1183/1188	658	1708	624	1752	—	—	—	—	—	—
Li et al.	2020	Chinese	1169/1277	1324	1014	1551	1003	375	574	220	470	611	196
Azarova et al.	2020	Russian	1579/1627	1988	1170	2204	1050	636	716	227	721	762	144
Tian et al.	2019	Chinese	508/493	506	510	570	416	130	246	132	171	228	94
Li et al.	2018	Chinese	123/311	128	118	327	295	34	60	29	94	139	78
Rao et al.	2016	Chinese	456/417	521	391	457	377	150	221	85	125	207	85
Unoki et al.	2008	Japanese	4795/3441	5119	4471	4019	2863	1431	2257	1107	1176	1667	598
Lu et al.	2012	Chinese	2899/3260	3157	2641	3868	2652	880	1397	622	1156	1556	548

n, Number; T2DM, type 2 diabetes mellitus; NDM, Non-diabetic subject (-), not applicable.

**TABLE 3:** Characteristics of each study included in rs9460546 of meta-analysis.

Author	Year	Ethnic	T2DM/NDM	ORs with 95% CI (G vs T)
Li et al.	2020	Chinese	1169/1277	1.133 (1.011–1.270)
Hu et al.	2009	Chinese	1849/1785	1.145 (1.041–1.260)
Herder et al.	2008	German	433/1438	1.410 (1.190–1.680)
Unoki et al.	2008	Japanese	4775/3442	1.226 (1.152–1.305)
Maller et al.	2012	European	632/677	1.250 (1.150–1.350)

T2DM, type 2 diabetes mellitus; NDM, Non-diabetic subject; OR, odds ratio; CI, confidence interval.

### 3.2 Heterogeneity Analysis

#### 3.2.1 rs4712523

**FIGURE 2:**
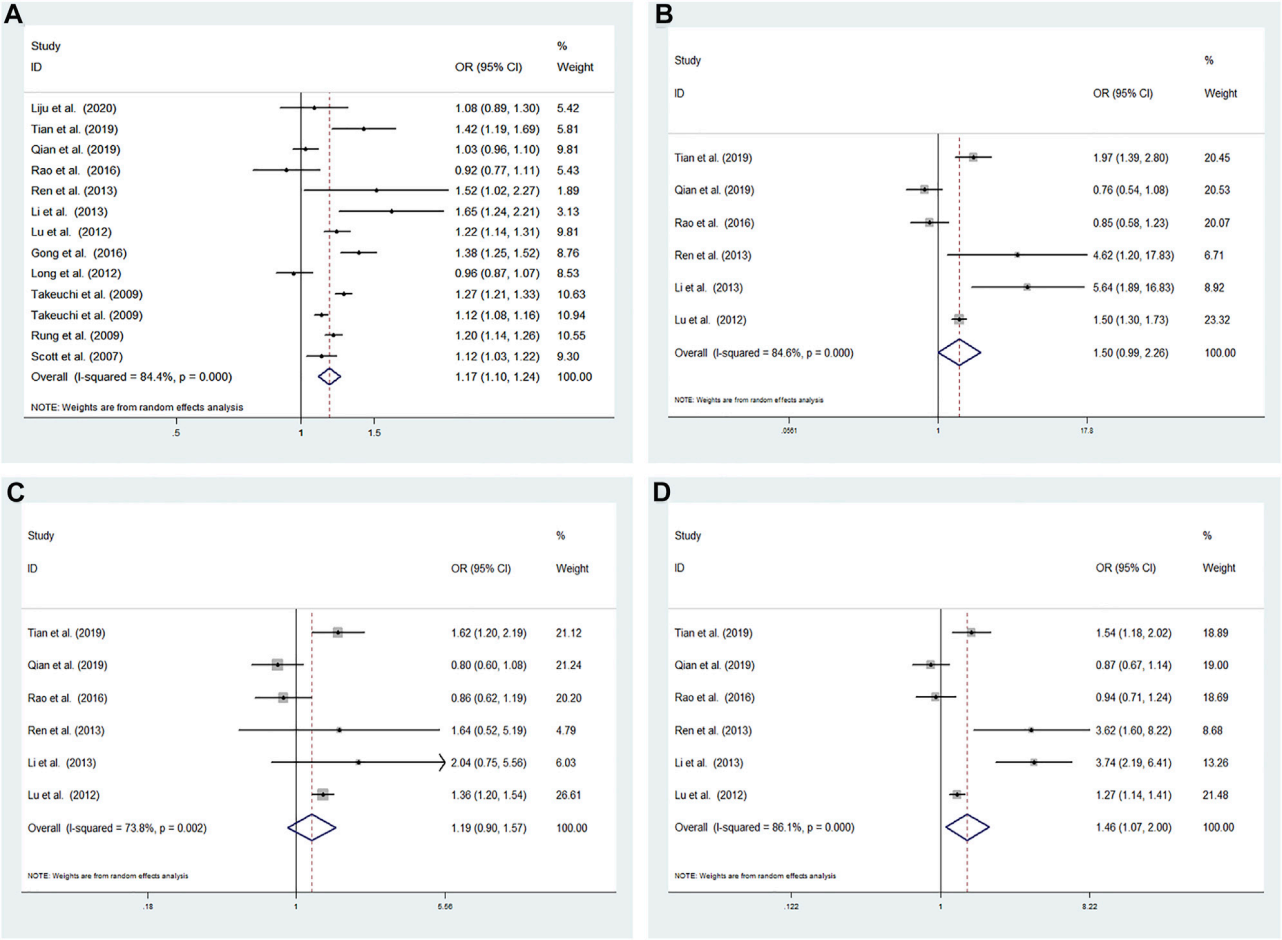
Meta-analysis using a random effects model for the association between the CDKALl rs4712523 polymorphism and T2DM susceptibility **(A)** Allele model, G vs A **(B)** Additive model, GG vs AA **(C)** Recessive model, GG vs AA + AG **(D)** Dominant model, GG + AG vs AA. OR: odds ratio, CI: confidence interval, I-squared: measure to quantify the degree of heterogeneity in meta-analyses.

High heterogeneity among studies (Scott et al., 2007; Rung et al., 2009; Takeuchi et al., 2009; Long et al., 2012; Lu et al., 2012; Gong, 2016; Li et al., 2013; Ren et al., 2013; Rao et al., 2016; Qian, 2019; Tian et al., 2019; Liju et al., 2020) was detected in the allele model (G vs A: I^2^ = 84.4%; *p* < 0.001), additive model (GG vs AA: I^2^ = 84.6%; *p* < 0.001), recessive model (GG vs AA + AG: I^2^ = 73.8%; *p* = 0.002), and dominant model (GG + AG vs AA: I^2^ = 86.1%; *p* < 0.001) (Figure 2).

#### 3.2.2 rs4712524

**FIGURE 3:**
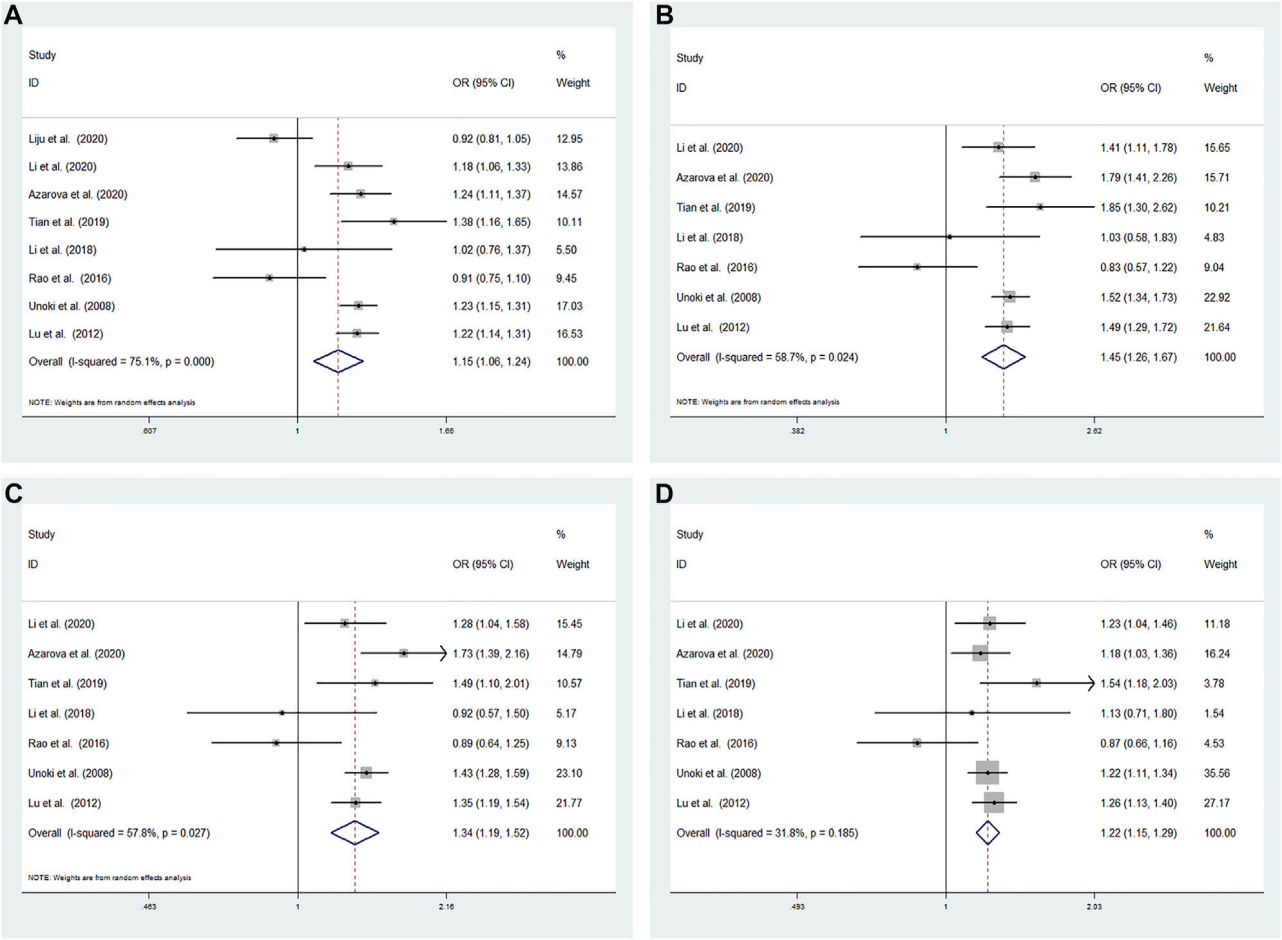
Meta-analysis for the association between the CDKALl rs4712524 polymorphism and T2DM susceptibility **(A)** Allele model, G vs A (random effects model) **(B)** Additive model, GG vs AA (random effects model) **(C)** Recessive model, GG vs AA + AG (random effects model) **(D)** Dominant model, GG + AG vs AA (fixed effects model). OR: odds ratio, CI: confidence interval, I-squared: measure to quantify the degree of heterogeneity in meta-analyses.

High heterogeneity among studies (Unoki et al., 2008; Lu et al., 2012; Rao et al., 2016; Li, 2018; Tian et al., 2019; Azarova, 2020; Li et al., 2020; Liju et al., 2020) was detected in the allele model (G vs A: I^2^ = 75.1%; *p* < 0.001). A moderate degree of heterogeneity among studies was detected under the additive model (GG vs AA: I^2^ = 58.7%; *p* = 0.024) and recessive model (GG vs AA + AG: I^2^ = 57.8%; *p* = 0.027). Low heterogeneity among studies was detected under the dominant model (GG + AG vs AA: I^2^ = 31.8%; *p* = 0.185) (Figure 3).

#### 3.2.3 rs9460546

**FIGURE 4:**
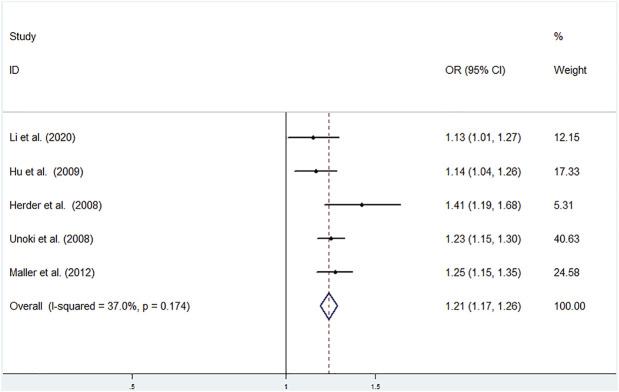
Meta-analysis using a fixed effects model for the association between the *CDKAL1* rs9460546 polymorphism and T2DM susceptibility (Allele model, G vs T). OR: odds ratio, CI: confidence interval, I-squared: measure to quantify the degree of heterogeneity in meta-analyses.

Low heterogeneity among studies (Herder et al., 2008; Unoki et al., 2008; Hu et al., 2009; Maller et al., 2012; Li et al., 2020) was detected in the allele model (G vs T: I^2^ = 37.0%; *p* = 0.174) (Figure 4).

### 3.3 Meta-Analysis Results

#### 3.3.1 rs4712523

A significant difference was found between T2DM patients and NDM controls for the allele model (G vs A: OR = 1.172; 95% CI: 1.103–1.245; *p* < 0.001) and dominant model (GG + AG vs AA: OR = 1.464; 95% CI: 1.073–1.996; *p* = 0.016). No significant associations were found under the additive model (GG vs AA: OR = 1.495; 95% CI: 0.990–2.257; *p* = 0.056) and recessive model (GG vs AA + AG: OR = 1.188; 95% CI: 0.900–1.568; *p* = 0.223) using a random effects model (Figure 2).

#### 3.3.2 rs4712524

A random effects model was used to analyze the allele, additive and recessive models, and the dominant model was analyzed using a fixed effects model. A significant difference was found between T2DM patients and NDM controls for the allele model (G vs A: OR = 1.146; 95% CI: 1.056–1.245; *p* = 0.001), additive model (GG vs AA: OR = 1.455; 95% CI: 1.265–1.673; *p* < 0.001) recessive model (GG vs AA + AG: OR = 1.343; 95% CI: 1.187–1.518; *p* < 0.001) and dominant model (GG + AG vs AA: OR = 1.221; 95% CI: 1.155–1.292; *p* < 0.001) (Figure 3).

#### 3.3.3 rs9460546

A significant difference was found between T2DM patients and NDM controls for the allele model (G vs T: OR = 1.215; 95% CI: 1.167–1.264; *p* = 0.023) using a fixed effects model (Figure 4).

### 3.4 Subgroup Analyses

#### 3.4.1 rs4712523

**FIGURE 5:**
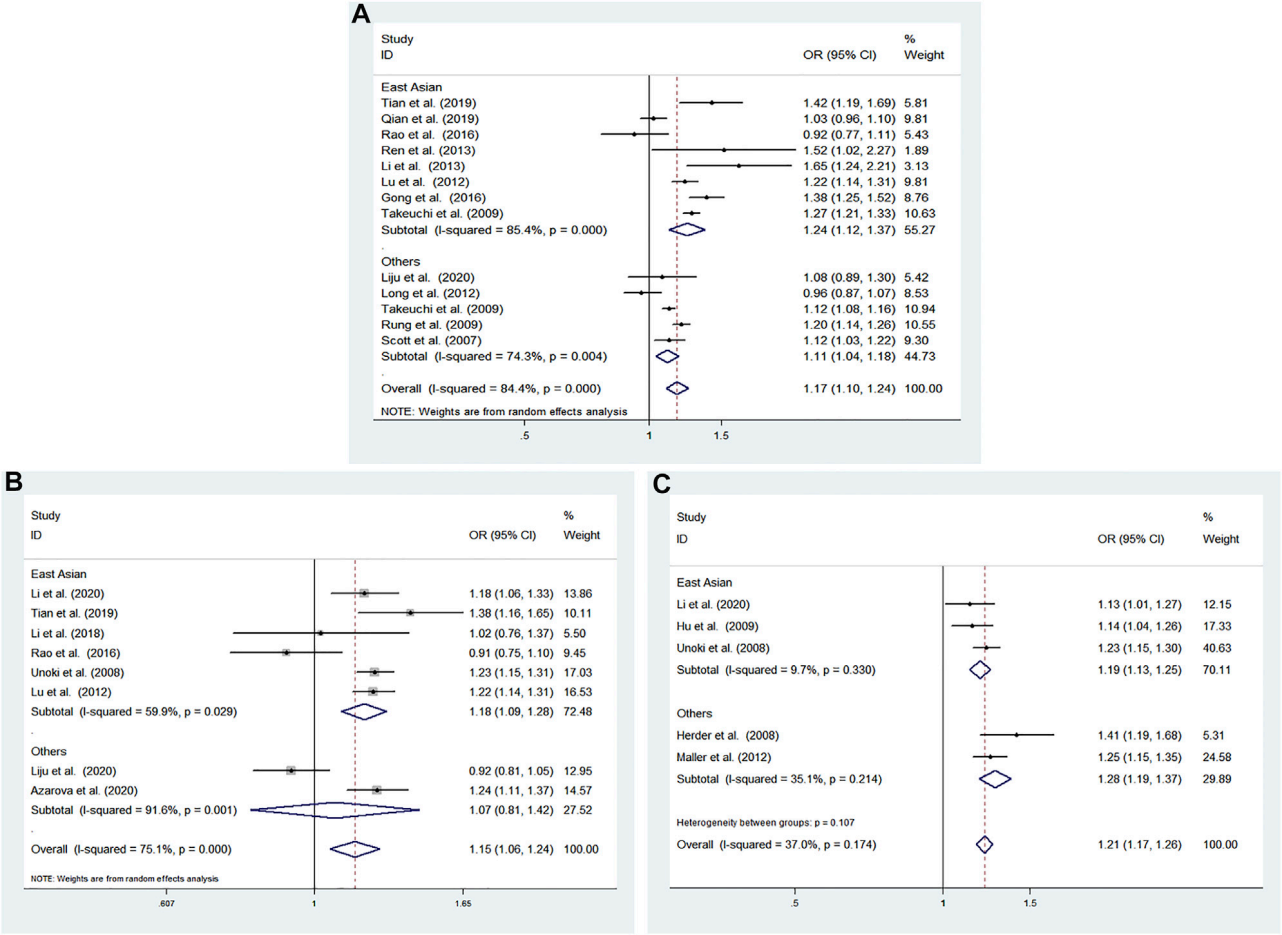
Association between the CDKALl variants and T2DM susceptibility in the subgroup for the allele model **(A)** rs4712523: G vs A (random effects model) **(B)** rs4712524: G vs A (random effects model) **(C)** rs9460546: G vs T (fixed effects model). OR: odds ratio, CI: confidence interval, I-squared: measure to quantify the degree of heterogeneity in meta-analyses.

We performed subgroup analysis according to ethnicity to evaluate the association between rs4712523 and T2DM susceptibility in the allele model. Rs35767 was significantly related to the risk of T2DM in the East Asian (G vs A: OR = 1.241; 95% CI: 1.123–1.371; *p* < 0.001) and others subgroup (G vs A: OR = 1.108; 95% CI: 1.039–1.180; *p* = 0.002) using a random effects model (Figure 5A).

#### 3.4.2 rs4712524

We performed subgroup analysis according to ethnicity to evaluate the association between rs4712524 and T2DM susceptibility in the allele model. Rs4712524 was significantly related to the risk of T2DM in the East Asian (G vs A: OR = 1.182; 95% CI: 1.095–1.277; *p* < 0.001), but no significant associations were found in others subgroup (G vs A: OR = 1.071; 95% CI: 0.807–1.423; *p* = 0.634) using a random effects model (Figure 5B).

#### 3.4.3 rs9460546

We performed subgroup analysis according to ethnicity to evaluate the association between rs9460546 and T2DM susceptibility in the allele model. Rs9460546 was significantly related to the risk of T2DM in the East Asian (G vs T: OR = 1.189; 95% CI: 1.134–1.247; *p* < 0.001) and others subgroup (G vs T: OR = 1.277; 95% CI: 1.188–1.373; *p* < 0.001) using a fixed effects model (Figure 5C).

### 3.5 Publication Bias

**FIGURE 6:**
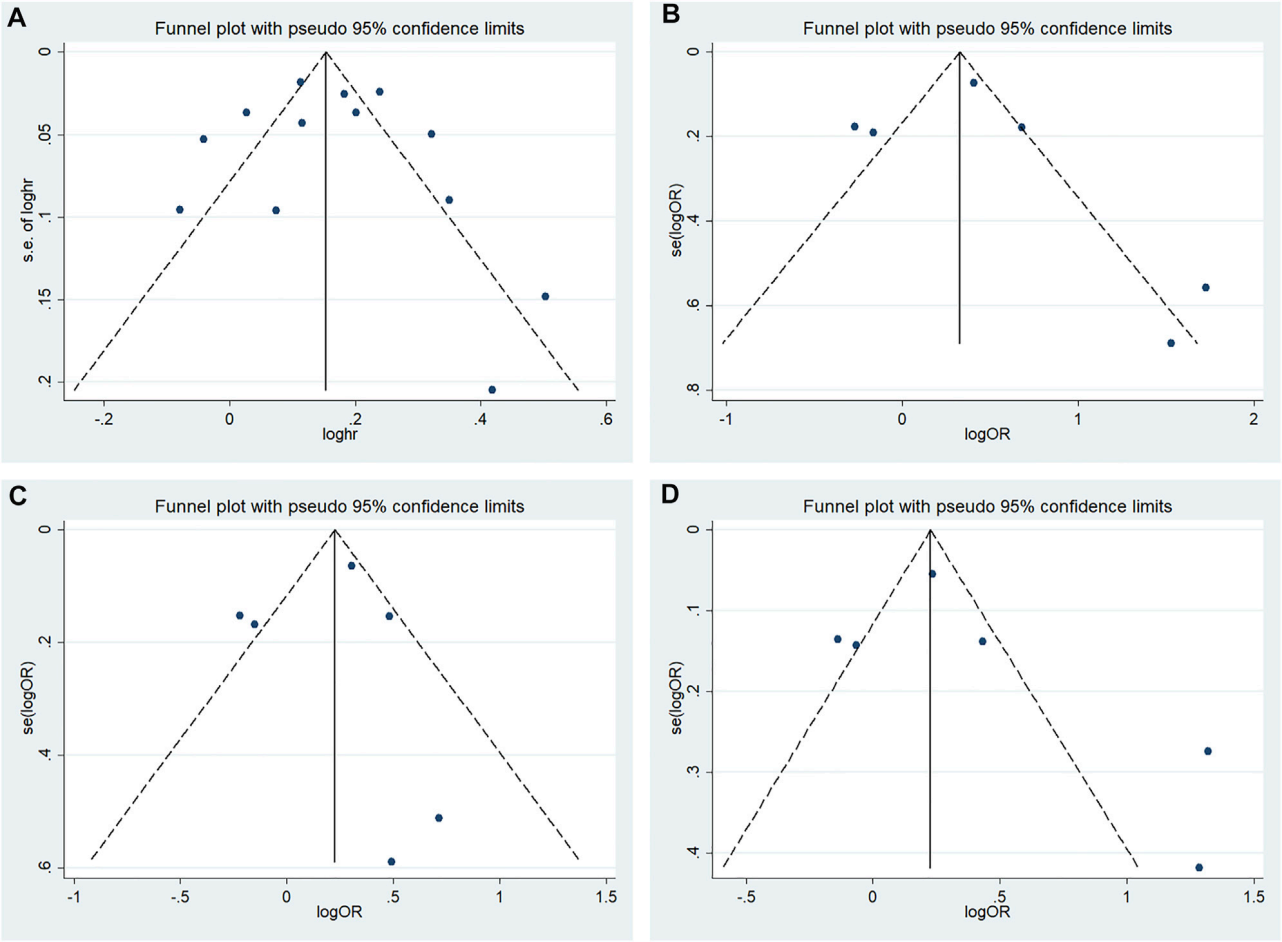
Funnel plot of the odds ratios in the CDKALl rs4712523 meta-analysis **(A)** Allele model, G vs A **(B)** Additive model, GG vs AA **(C)** Recessive model, GG vs AA + AG **(D)** Dominant model, GG + AG vs AA.

**FIGURE 7:**
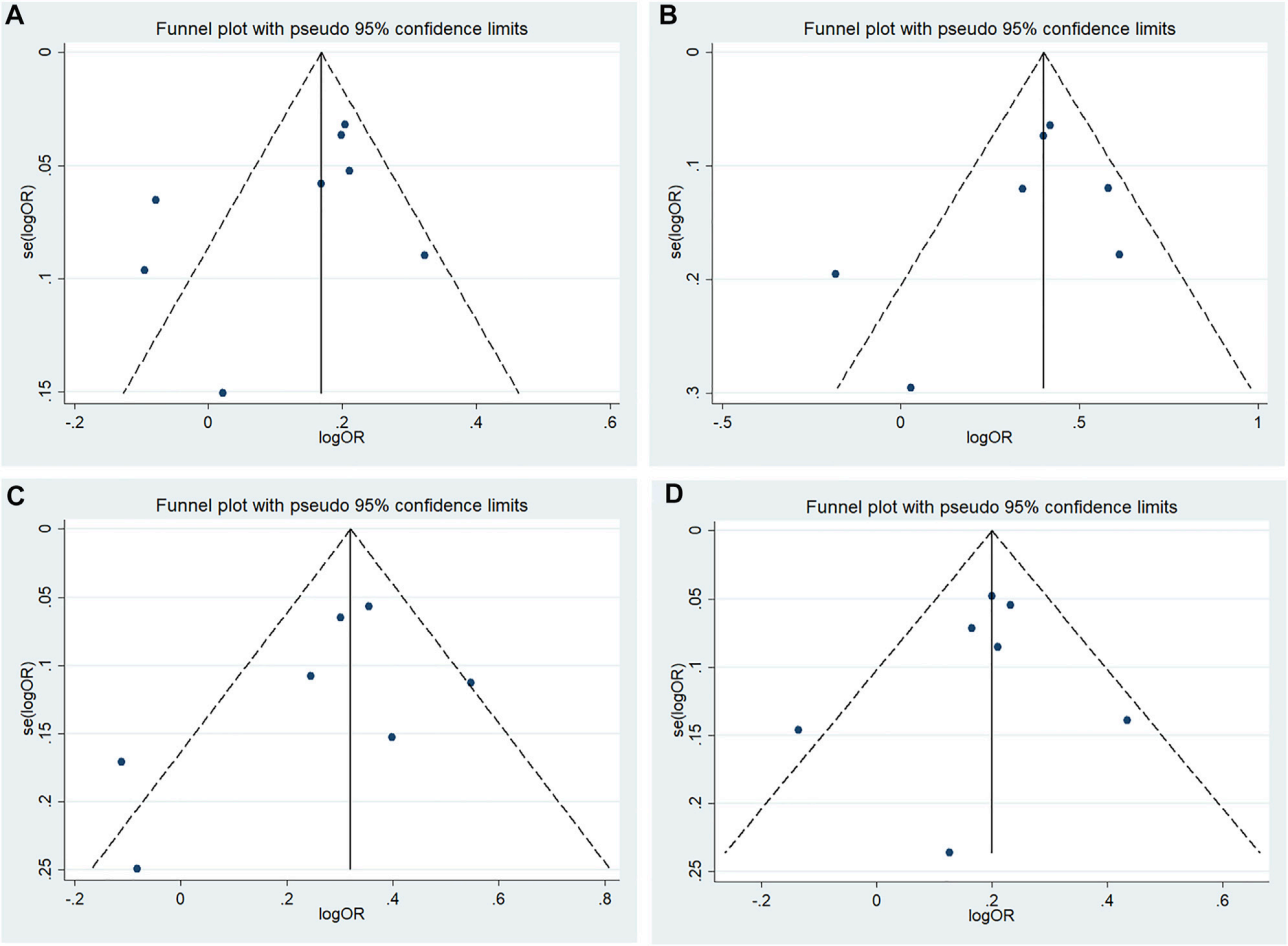
Funnel plot of the odds ratios in the CDKALl rs4712524 meta-analysis **(A)** Allele model, G vs A **(B)** Additive model, GG vs AA **(C)** Recessive model, GG vs AA + AG **(D)** Dominant model, GG + AG vs A.

**FIGURE 8:**
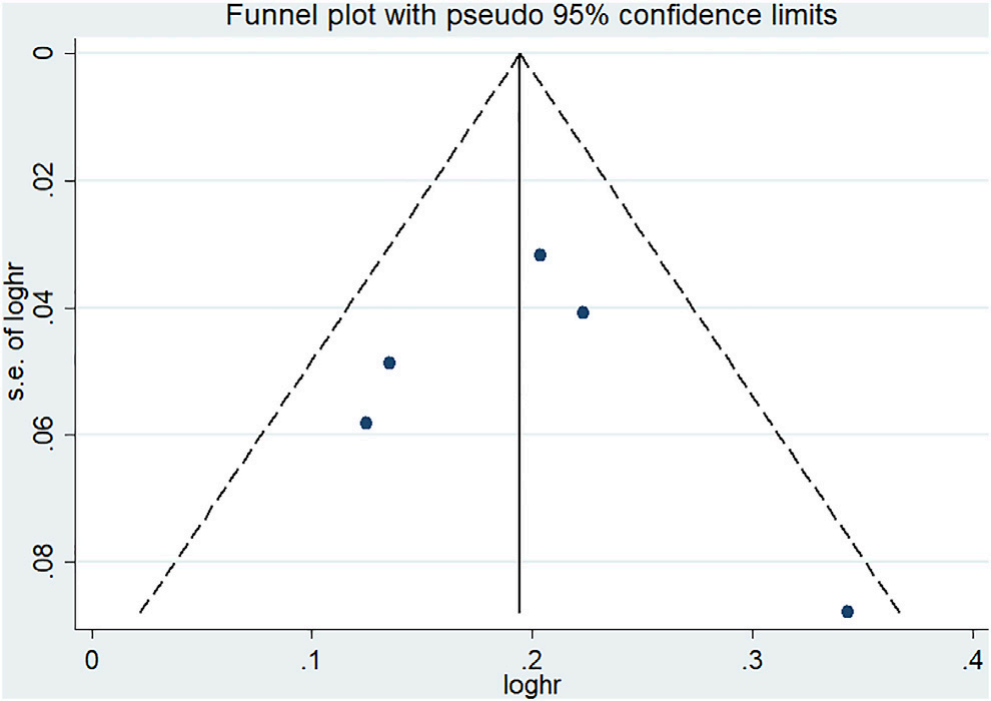
Funnel plot of the odds ratios in the CDKALl rs9460546 meta-analysis for the allele model (G vs T).

**FIGURE 9:**
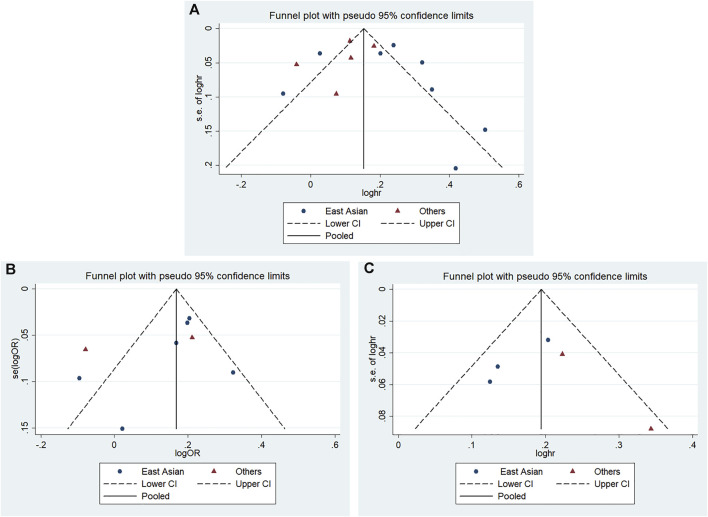
Funnel plot of the odds ratios in the CDKALl variants in the subgroup meta-analysis for the allele model **(A)** rs4712523: G vs A **(B)** rs4712524: G vs A **(C)** rs9460546: G vs T.

According to Begg’s and Egger’s tests, no significant publication bias was found in each of the genetic models (all *p* > 0.05, data not shown), and the funnel plots are shown in Figures 6–9.

## 4 Discussion

*CDKAL1* is a key pathogenesis-related protein for T2DM (Tian et al., 2019). Genetic variants may play an essential role in T2DM susceptibility. In this meta-analysis, three SNPs (rs4712523, rs4712524, and rs9460546) from previous studies were evaluated to determine the association of *CDKAL1* polymorphisms with T2DM. *CDKAL1* polymorphisms (rs4712523, rs4712524, and rs9460546) showed a significant association with T2DM. Our results were consistent with some previous study findings.

The results revealed that the G allele and GG + AG genotypes of rs4712523 were associated with an increased risk of T2DM. Nine of the thirteen previous studies investigated rs4712523 showed an association between the G allele and T2DM (Scott et al., 2007; Rung et al., 2009; Takeuchi et al., 2009; Long et al., 2012; Lu et al., 2012; Gong, 2016; Li et al., 2013; Ren et al., 2013; Tian et al., 2019), and four studies found an association between the GG + AG genotypes and T2DM (Lu et al., 2012; Li et al., 2013; Ren et al., 2013; Tian et al., 2019). In addition, the rs4712524 G allele, GG and GG + AG genotypes were associated with an increased risk of T2DM susceptibility. That have been confirmed previous observations (Unoki et al., 2008; Lu et al., 2012; Tian et al., 2019; Azarova, 2020; Li et al., 2020). Additionally, the results showed that rs9460546 G allele was associated with T2DM susceptibility. Markedly, all five studies found that the rs9460546 G allele was associated with T2DM in various populations (Herder et al., 2008; Unoki et al., 2008; Hu et al., 2009; Maller et al., 2012; Li et al., 2020). Moreovr, rs4712523, rs4712524, and rs9460546 showed a significant association with T2DM in the East Asian subgroup for the allele model. In general, Our results have confirmed previous observations suggesting that *CDKAL1* may play a role in T2DM. But it is worth noting that high heterogeneity among studies was detected in rs4712523 and rs4712524 likely because of the difference in country, ethnicity, genetic background and environmental factors. Subgroup analyses were performed by ethnicity in the allele model, and the subgroup still had high heterogeneity. Importantly, the high heterogeneity among studies might have affected our data.

*CDKAL1* expression in human pancreatic β-cells increases insulin secretion by inhibiting CDK5 (Li et al., 2020; Wei et al., 2005; Ubeda et al., 2006; Ching et al., 2002). Subsequently, several studies have shown the association of genetic variants in *CDKAL1* with defects in proinsulin conversion and the insulin response following glucose stimulation (Pascoe et al., 2007; Steinthorsdottir et al., 2007; Tian et al., 2019). Thus, *CDKAL1* is involved in the development of T2DM. Genome-wide association studies have identified several SNPs in the *CDKAL1* gene associated with T2D (Saxena et al., 2007; Scott et al., 2007; Tian et al., 2019). Our results confirmed the significant association between *CDKAL1* SNPs and T2DM susceptibility. However, the mechanisms must be verified in functional studies. Our association results provide reference data to identify new biomarkers of T2DM that could contribute to the diagnosis of T2DM.

This meta-analysis has a few limitations. First, because of the limited examination of *CDKAL1* variants in T2DM, the included studies had comparatively small sample sizes, which might affect the results of the meta-analysis because of insufficient statistical power. Thus, studies must be performed across different geographical and ethnic groups. Additionally, the factors of T2DM might be complex, with the contribution of genetic, environmental and dietary habits. Therefore, further study is required to evaluate whether other risk factors together with the *CDKAL1* gene influence T2DM susceptibility.

## 5 Conclusion

To our knowledge, this study is the first to assess the role of *CDKAL1* polymorphisms (rs4712523, rs4712524, and rs9460546) in T2DM. Significant associations were found between the *CDKAL1* rs4712523, rs4712524, and rs9460546 polymorphisms and susceptibility to T2DM.

## Data Availability

The original contributions presented in the study are included in the article/supplementary material, further inquiries can be directed to the corresponding authors.
